# A Very Rare Gun-Shot Wound

**Published:** 1862-06

**Authors:** Charles G. Smith


### A VERY RARE CASE OF GUN-SHOT WOUND.

### BALL PASSING IN THROUGH THE THORAX AND COMING OUT AT THE ANUS.

Reported by CHARLES G. SMITH, M.D.

On board the steamer War Eagle, which conveyed about 400 of the solders wounded at the Battle of Shiloh, from Pittsburg Landing to Cincinnati, and to which steamer I was detailed by the Medical Director at Pittsburg, I found the following case:

Corporal George Hipwell, of the 15th Ill. Volunteers, had been wounded on the first day of the battle, Sunday, April 6th, 1862. The ball passed into the thorax, under the right scapula, between the fifth and sixth ribs, and disappeared. He raised a good deal of blood, he said, for three or four days after he was wounded, but whether by coughing or vomiting I could not make out from his description. When I first saw him, which was five days after the battle, he was a good deal depressed; but he began to improve gradually under a moderate use of stimulants and an improved quality of food. On Sunday, however, just one week from the time he was shot, while sitting at stool, over the pail, he suddenly jumped up, exclaiming that he had passed something strange, and found the bullet in his passage. This was testified to by the nurse, John Lindars, of the same company, who was holding him over the bucket, and who was sure that the pail was entirely empty before the patient took his seat upon it. The ball was not a Minnie, but one of the old fashioned round ones. It was a little dented, from coming in contact with the ribs, probably.

From that time he recovered rapidly, and was doing very well when I left him in Cincinnati. I have thought the case so unusual that it would interest the Medical Society to-night, and I am happy to state that I have lately heard from the patient, and he is, at this date, June 7th, just two months from the accident, doing well, and suffering no further inconvenience from his strange wound.

